# Levels of Soluble Endothelium Adhesion Molecules and Complications among Sickle Cell Disease Patients in Ghana

**DOI:** 10.3390/diseases6020029

**Published:** 2018-04-21

**Authors:** Charles Antwi-Boasiako, Eric S. Donkor, Fredericka Sey, Bartholomew Dzudzor, Gifty B. Dankwah, Kate H. Otu, Alfred Doku, Campbell A. Dale, Ivy Ekem

**Affiliations:** 1 Department of Physiology, School of Biomedical and Allied Health Sciences, University of Ghana, Accra, Ghana; gdankwah@gmail.com; 2 Department of Medical Microbiology, School of Biomedical and Allied Health Sciences, University of Ghana, Accra, Ghana; ericsdon@hotmail.com; 3 Sickle Cell Clinic, Korle-Bu Teaching Hospital, Accra, Ghana; fredisey@hotmail.com; 4 Department of Medical Biochemistry, School of Biomedical and Allied Health Sciences, University of Ghana, Accra, Ghana; dzudzorb@yahoo.com; 5 Department of Nursing and Midwifery, Greenhills School of Health Sciences, Accra, Ghana; kateboasiako@yahoo.co.uk; 6 Department of internal Medicine, School of Medicine and Dentistry, University of Ghana, Accra, Ghana; dokukavin@gmail.com; 7 Department of Pediatric Hematology/Oncology, University of Michigan Hospitals, Ann Arbor, MI 48109, USA; acampbell@childrensnational.org; 8 Department of Haematology, School of Medical Sciences, College of Health and Allied Sciences, University of Cape Coast, Cape Coast, Ghana; ekem_ivy@hotmail.com

**Keywords:** sickle cell disease, endothelial dysfunction, soluble adhesion molecules, ICAM-1, VCAM-1 and E-Selectin

## Abstract

Background: Soluble adhesion molecules are involved in the gathering and joining of inflammatory cells to vascular endothelium. Therefore, they serve as potential markers of endothelial dysfunction in vascular diseases including sickle cell disease (SCD). In Ghana, there are scarcely any report on the levels of adhesion molecules among SCD patients. The current study aimed to determine plasma levels of ICAM-1, VCAM-1 and E-Selectin as markers of endothelial dysfunction in SCD patients in steady state, complications and controls. Methodology: This was a cross-sectional study involving 60 HbAA controls, 46 HbSS steady state, 57 HbSS VOC, 18 HbSC VOC, 21 HbSS with leg ulcer and 11 HbSS with priapism. Blood samples were collected from all the study subjects (*n* = 213) and processed into plasma. The plasma levels of VCAM-1, ICAM-1 and E-Selectin concentrations of SCD patients and controls were measured using a double sandwich ELISA technique. Demographic information was also collected from the study subjects. Results: Levels of all soluble proteins (ICAM-1, VCAM-1 and E-Selectin) were significantly higher in HbSS steady-state patients compared to non-SCD controls (*p* < 0.001). Generally, SCD patients with complications had relatively higher levels of the soluble proteins compared to those in the steady-state. Of the SCD patients with complications, those with vaso-occlusion crisis (HbSS VOC) had relatively higher levels of ICAM-1, VCAM-1 and E-Selectin at (62.42 ng/mL ± 26.09), (634.99 ng/mL ± 324.31) and (236.77 ng/mL ± 114.40) respectively; Conclusion: Although levels of adhesion molecules were high in all the SCD patients with complications, those with vaso-occlusive crisis had higher levels. This might reflect an ongoing endothelial dysfunction in these patients. SCD patients with vaso-occlusive crisis presents with a more severe pathophysiology condition.

## 1. Introduction

Sickle Cell Disease (SCD) refers to a number of genetic disorders associated with structurally abnormal haemoglobin, resulting in the episodic formation of sickle-shaped red blood cells and several clinical manifestations. The underlying genetic abnormality is a point mutation (GTG for GAG) in the gene for β-globin on chromosome 11, leading to the replacement of a glutamic acid residue with valine on the surface of the protein (termed HbS) [1]. The SCD is one of the most common monogenetic disorders in the world, affecting nearly 1 in 600 African Americans [2], and an estimated 1 to 4% of babies born in sub-Saharan Africa [3]. The predominant genotypes that give rise to SCD include Hb SS, Hb SC, Hb Sβ^+^-thalassemia and Hb Sβ^0^-thalassemia; other rare forms include hemoglobin SD and hemoglobin SE [4]. Complications from sickle cell disease can include gallstones, lung crises (acute chest syndrome), pulmonary hypertension, stroke, leg ulcers that do not heal, and eye damage [5]. The most common and frequently reported complications of SCD is renal dysfunction [6].

Endothelial dysfunction is associated specifically with anticoagulant properties, increased platelet aggregation, increased expression of vascular adhesion molecules (VCAM-1, ICAM-1 and E-Selectin), increased expression of chemokines and cytokines as well as increased reactive oxygen species production from the endothelium [7]. An important characteristic of endothelial dysfunction is the inability of arteries and arterioles to optimally dilate in response to an appropriate stimulus by vasodilators acting on the endothelium [8]. Soluble endothelial cell adhesion molecules (ICAM-1, VCAM-1 and E-Selectin) play a very important physiological role in the recruitment and binding of inflammatory cells to vascular endothelium, particularly in venules [9]. These potential markers of endothelial dysfunction are characterized by endothelial activation [10], and may play an important role in the development of SCD complications including VOC, leg ulcers and priapism [11]. E-Selectin induce specific inflammatory cells to slowly move across the surface of the endothelial, until a strong adhesive interaction develop on VCAM-1 as well as ICAM-1 to specific counter-ligands on the surface of inflammatory cells [9].

Levels of soluble VCAM-1 (sVCAM-1) have been shown to rise in the plasma of SCD patients. SCD patients have increased expression of adhesion molecules such as VCAM-1, ICAM-1 and E-Selectin during VOC and asymptomatic periods. Thus, a reflection of ongoing endothelial activation and damage is useful [12]. In Ghana, studies have been done in the area of epidemiology of SCD and its complications [13], but there is no report on the possible association between endothelial cell adhesion molecules (VCAM-1, ICAM-1 and E-Selectin) and endothelial dysfunction in SCD patients. Investigating the levels of these adhesion molecules in SCD patients with a hemolytic clinical sub-phenotype that includes priapism and leg ulcers may help in the management of SCD, as they may serve as potential biomarkers in these patients. The present study was aimed at determining plasma levels of ICAM-1, VCAM-1 and E-Selectin as markers of endothelial dysfunction in SCD patients in steady state, complications and controls.

## 2. Methods

### 2.1. Study Site, Design and Sampling

This was a cross-sectional study conducted at the Canter for Clinical Genetics (sickle cell clinic) of the Korle-Bu Teaching hospital (KBTH), Accra, Ghana from February 2013 to May 2015. The population of Ghana is about 25 million [14], and the prevalence of Sickle Cell Disease is 1.9% of all births per year [15]. The out-patient morbidity reports compiled by the Ghana Health Service in 2002 and 2003 ranked SCD at the 37th and 36th positions, respectively [16]. KBTH is the largest hospital in Ghana with a bed capacity of about 2000 and 17 clinical and diagnostic departments including a Sickle Cell Clinic [17]. The hospital has an average daily attendance of 1500 patients and about 250 patient admissions [17]. Based on 95% confidence level and 5% allowable error, 213 SCD patients were randomly sampled at the Sickle Cell Clinic of KBTH including a control group of 60 HbAA recruited from the National Blood Transfusion Centre located at KBTH. Patients with conditions such as diabetes mellitus, hypertension, coronary artery disease, renal failure, pregnancy and recent blood transfusion (three months prior to the study) were excluded from the study. The study protocol was approved by the Ethical and Protocol review Committee of University of Ghana Medical School. Written informed consents were obtained from participants/study subjects before samples were obtained. The determination of haemoglobin genotypes of the study participants was based on haemoglobin electrophoresis. Five milliliters (5 mls) of venous blood sample was collected from each of the study participants into ethylene diamine tetracetic acid (EDTA) tubes for analysis. Vaso-occlusive crisis was clinically defined as pains in the bones, muscles and joints that are not due to any other cause and requiring parenteral analgesia and thus, admitted in the Centre for some hours. Steady state was defined clinically, as a patient who has been well and has not been in any crisis for at least two weeks. Leg ulcer was defined as a defect in the skin below the level of the knee and above the foot, which persists for six weeks or more. Priapism was also defined as a purposeless, persistent penile erection, particularly unaccompanied by any stimulation or sexual desire, which usually last for more than 6 h.

### 2.2. Laboratory Analysis

Aliquot of 2.5 mls of the blood sample was processed into plasma and stored at −80 °C. The plasma levels of VCAM-1, ICAM-1 and E-Selectin concentrations of HbSS SCD patients and controls were measured by using human VCAM-1, ICAM-1 and E-Selectin ELISA kits respectively (GenWay, San Diego, CA, USA, ELISA Development kit) according to the manufacturer‘s instructions.

Standard human VCAM-1 solutions of concentrations 10,000 pg/mL, 5000 pg/mL, 2500 pg/mL, 1250 pg/mL, 625 pg/mL, 313 pg/mL and 156 pg/mL were prepared from the 10 ng stock using the sample diluent buffer provided. Aliquots of 0.1 mL of each standard were added to the 96-well pre-coated plate in duplicate and 0.1 mL of the sample diluent buffer into the control well. A volume of 0.1 mL of the sample plasma (diluted 1:100 with sample diluent buffer) was added into the remaining wells in duplicate. The plate was sealed with cellophane and incubated at 37 °C for 90 min. After the incubation period, the plate content was discarded and the plate blotted with a paper towel.

Aliquots of 0.1 mL biotinylated anti-human ICAM-1 antibody working solution were added into each well and the plate incubated at 37 °C for 60 min. The plate was then washed 3 times with 0.01 M phosphate buffered saline (PBS) using an automated plate washer. The washing buffer was then discarded and the plate blotted dry on paper towels. A volume of 0.1 mL of prepared Avidin-Biotin-Peroxidase Complex (ABC) working solution was added into each well and the plate incubated at 37 °C for 30 min. The plate was then washed 5 times with 0.01M PBS and blotted onto paper towels. After this, 90 μL of prepared TMB color developing agent was added into each well and the plate incubated at 37 °C in the dark for 25 min. 0.1 mL of prepared TMB stop solution was then added into each well. The optical density absorbance was then read at 450 nm in a microplate reader (Amersham Bioscience Limited, Buckinghamshire, UK).

For assay of E-Selectin, the procedure was repeated using Standard human E-Selectin solutions of concentrations 10,000 pg/mL, 8000 pg/mL, 4000 pg/mL, 2000 pg/mL, 1000 pg/mL, 500 pg/mL, 250 pg/mL and 125 pg/mL and 0.1 mL biotinylated anti-human E-Selectin antibody working solution.

### 2.3. Data Analysis

The data was entered in to SPSS version-20 software. Frequency tables were generated for nominal and ordinal variables. The results were expressed as mean plus or minus standard deviation (mean ± SD). The Kruskal Wallis test was used to compare differences in mean values among SCD patients in steady state and VOC, as well as those with leg ulcer and priapism with the healthy controls. Dunn’s test was done as a post hoc analysis for multiple comparisons. Statistical significance was considered at *p* < 0.05.

## 3. Results

### 3.1. Demographic and Clinical Features of the Study Participants

The 213 enrolled subjects recruited in this study included 60 HbAA apparently healthy controls (30 males, 30 females), and 153 SCD patients (83 males, 70 females). The mean age of the HbAA controls was 31.9 ± 10.0 years and 30.5 ± 9.7 years for the SCD group. Thirty percent SCD patients were in steady state (*n* = 46), 49% were in active VOC (*n* = 75), 14% (*n* = 21) had an active leg ulcer, and 7% (*n* = 11) had an active priapism. Males had twice as many leg Ulcers (*n* = 14) than females (*n* = 7) in symptomatic patients. However, females (*n* = 45) comprised of the majority of VOC (45 vs. 30 males) patients.

### 3.2. Plasma ICAM-1, VCAM-1 and E-Selectin Levels in Controls and SCD Patients with and Without Complications

Soluble protein levels in all SCD group and non-SCD controls are shown in Table 1. Levels of all soluble proteins (ICAM-1, VCAM-1 and E-Selectin) were significantly higher in HbSS steady-state patients compared to non-SCD controls (*p* < 0.001). The levels were further increased in HbSS VOC patients. There was no increase in all protein levels in leg ulcer HbSS compared to steady-state HbSS patients. There was increase in ICAM-1 level in HbSS priapism patients compared to HbSS steady-state patients (*p* < 0.001). Compared to HbSS leg ulcers, subjects with HbSS priapism had significantly higher ICAM levels (*p* < 0.001).

## 4. Discussion

This study highlights the involvement of adhesion molecules (VCAM-1, ICAM-1, and E-Selectin) in the endothelial dysfunction of SCD patients with and without complications. The detection of relatively higher plasma levels of VCAM-1, ICAM-1, and E-Selectin in SCD patients suggest that, adhesion molecules play a vital physiological role in the recruitment and binding of inflammatory cells to vascular endothelium [9]. Thus, it is not surprising that the levels of VCAM-1, ICAM-1, and E-Selectin recorded were even higher in SCD patients with VOC. In line with another study [12], increased VCAM-1, ICAM-1, and E-Selectin were seen in SCD patients during the vaso-occlusive state. Report from previous studies conducted elsewhere suggests that, SCD involves an abnormally activated, pro-adhesive endothelial cell state and demonstrate increased expression of the adhesion molecules [18,19,20]. The present study highlights a similar trend in levels of adhesion molecules (VCAM-1, ICAM-1, and E-Selectin) among patients with SCD. Higher levels of VCAM-1, ICAM-1, and E-Selectin in SCD patients are usually coupled with significantly low plasma NO levels, derived from the stimulating action of various biological modifiers such as hypoxia, thrombin, and cytokines on the normally quiescent endothelium [19], as well as from the pro-inflammatory effects of reperfusion-injury physiology [21,22]. Thus, higher levels of VCAM-1, ICAM-1, and E-Selectin are expected in SCD associated complications due to a possible activation of inflammatory cells in these patients, as observed in the present study.

Impaired NO production, which leads to decreased bioavailability of NO due to higher levels of these adhesion molecules could result in endothelial dysfunction [23,24]. This partly explains the higher levels of adhesion molecules observed in all the SCD patients recruited. Besides, a possible association between plasma levels of VCAM-1, ICAM-1, and E-Selectin, and endothelial dysfunction, exists in SCD patients. In line with other similar studies [18,20], soluble levels of the adhesion molecules were lower in the controls. The observed increase in VCAM-1 and ICAM-1 levels in SCD patients probably reflects an increased level of endothelial cell adhesion molecule expression and activation in these patients as well as an increased capacity for the adhesion of sickle erythrocytes and leukocytes to the endothelium [20]. The view of SCD as a state of abnormal endothelial activation could present a potential opportunity for novel therapeutic approaches in that pharmacologic inhibition of endothelial cell activation might be clinically beneficial. This is because VCAM-1, ICAM-1, and E-Selectin are all markers of endothelial dysfunction, and have been implicated in the pathology of SCD [25,26]. Higher levels of E-Selectins demonstrated in the plasma of patients with SCD in both steady state and during VOC agree with the work of Najjar et al. [27] in patients of Saudi Arabia ethnicity and non-Saudi patients.

Increased levels of soluble (s) VCAM-1 and sICAM-1 have been reported to be associated with increased haemolytic rate in patients with SCD [28,29]. The similar levels of soluble endothelial adhesion molecules observed in both HbSS patients in steady state and those with leg ulcer could partly be due to a similar degree of hemolysis in these patients. Sub-phenotypes of SCD including priapism and leg ulcers are associated with increased hemolysis [30,31], a decrease in the NO precursor, L-arginine [32,33], reduced NO bioavailability [34], and a disruption of the coagulation cascade [35]. Therefore, a combination of each of these processes may play a significant role in the upregulation of adhesion molecule expression [35]. Nevertheless, soluble ICAM-1, VCAM-1 and E-Selectin may serve as promising biomarkers of pain in VOC, owing to the continuous increase in levels of all endothelial adhesion molecules in the current study. Higher levels of ICAM-1, VCAM-1 and E-Selectin might be very useful in predicting patients at high risk of endothelial dysfunction [10], especially in the case of SCD patients with VOC, in this study.

Our study had some limitations. We could not gather information on bilirubin, liver function tests, LDH, reticulocyte count, the percentage of patients on hydroxyurea and other environmental factors such as smoking and BMI, which may as well affect endothelial function.

## 5. Conclusions

Elevated levels of VCAM-1, ICAM-1 and E-Selectin were generally found in SCD patients, as compared to the control group in the present study. Elevated levels of soluble ICAM-1, VCAM-1 and E-Selectin found in Ghanaian HbSS patients in steady-state may reflect universal endothelial injury in SCD patients. Increased levels of ICAM-1, VCAM-1 and E-Selectin in HbSS VOC demonstrated further increase of endothelial injury associated with this complication. HbSS leg ulcer and priapism were not associated with further increase of endothelial injury in SCD patients. 

## Figures and Tables

**Table 1:** Plasma ICAM-1, VCAM-1 and E-Selectin levels in controls and SCD patients with and without complications.

Parameter	HbAA Control (*n* = 60)	HbSS Steady State (*n* = 46)	HbSS VOC (*n* = 57)	HbSC VOC (*n* = 18)	HbSS leg ulcer (*n* = 21)	HbSS Priapism (*n* = 11)	*p*-Value
ICAM–1 (ng/mL)	29.60 (12.03–40.32)	48.09 (24.72–70.14)	62.42 (26.09–62.42)	31.67 (13.56–68.94)	45.00 (17.42–95.06)	61.13 (34.46–81.18)	<0.001
VCAM–1 (ng/mL)	286.10 (179.36–356.21)	490.10 (314.45–980.15)	634.99 (324.31–934.69)	540.32 (258.73–876.37)	430.74 (143.48–739.75)	455.56 (374.24–852.49)	<0.001
E–selectin (ng/mL)	157.49 (138.96–543.53)	227.87 (119.68–624.46)	236.77 (114.40–632.50)	219.44 (92.51–829.49)	228.94 (94.83–721.96)	193.12 (70.24–562.18)	<0.001
